# Sleep-Wake Sensitive Mechanisms of Adenosine Release in the Basal Forebrain of Rodents: An *In Vitro* Study

**DOI:** 10.1371/journal.pone.0053814

**Published:** 2013-01-11

**Authors:** Robert Edward Sims, Houdini Ho Tin Wu, Nicholas Dale

**Affiliations:** School of Life Sciences, University of Warwick, Coventry, West Midlands, United Kingdom; Hôpital du Sacré-Coeur de Montréal, Canada

## Abstract

Adenosine acting in the basal forebrain is a key mediator of sleep homeostasis. Extracellular adenosine concentrations increase during wakefulness, especially during prolonged wakefulness and lead to increased sleep pressure and subsequent rebound sleep. The release of endogenous adenosine during the sleep-wake cycle has mainly been studied *in vivo* with microdialysis techniques. The biochemical changes that accompany sleep-wake status may be preserved *in vitro*. We have therefore used adenosine-sensitive biosensors in slices of the basal forebrain (BFB) to study both depolarization-evoked adenosine release and the steady state adenosine tone in rats, mice and hamsters. Adenosine release was evoked by high K^+^, AMPA, NMDA and mGlu receptor agonists, but not by other transmitters associated with wakefulness such as orexin, histamine or neurotensin. Evoked and basal adenosine release in the BFB *in vitro* exhibited three key features: the magnitude of each varied systematically with the diurnal time at which the animal was sacrificed; sleep deprivation prior to sacrifice greatly increased both evoked adenosine release and the basal tone; and the enhancement of evoked adenosine release and basal tone resulting from sleep deprivation was reversed by the inducible nitric oxide synthase (iNOS) inhibitor, 1400 W. These data indicate that characteristics of adenosine release recorded in the BFB *in vitro* reflect those that have been linked *in vivo* to the homeostatic control of sleep. Our results provide methodologically independent support for a key role for induction of iNOS as a trigger for enhanced adenosine release following sleep deprivation and suggest that this induction may constitute a biochemical memory of this state.

## Introduction

Sleep is a homeostatically-regulated process. Prolonged wakefulness leads to increased sleep pressure and consequent increased duration and intensity of sleep. The endogenous somnogen adenosine is a key mediator of sleep homeostasis. Adenosine or adenosine receptor agonists enhance sleep [1]–[5]. Adenosine receptor antagonists such as theophylline and caffeine are known stimulants that prevent sleep (for a review see [6]).The levels of adenosine increase throughout the brain during wakefulness and decrease during sleep, and this is particularly notable in the basal forebrain (BFB), part of the ascending arousal system [7]–[9]. Consequently, identifying the mechanisms of adenosine production, release and activity are vital to understanding its role in sleep homeostasis.

McCarley and colleagues [10] proposed that adenosine in the basal forebrain acting via adenosine A_1_ receptors inhibited the cholinergic neurons of the BFB which provide input to the cerebral cortex. However, despite the evidence for the role played by A_1_ receptors [11]–[14] there may be additional mechanisms involved. A_2a_ receptors are also implicated in sleep as A_1_ receptor null mice have normal sleep patterns and exhibit rebound sleep similar to wild type [15], nor are affected by caffeine [16].

Nitric oxide (NO) signaling has been proposed as a trigger for enhanced adenosine release during sleep deprivation [17]. Delivery of 1400 W (an antagonist selective for inducible nitric oxide synthase (iNOS; [18]), L-NAME (a general NOS inhibitor) and cPTIO (a NOS scavenger; [17]) through microdialysis into the BFB all inhibited the increase extracellular adenosine concentrations in BFB that follows sleep deprivation. Furthermore, both the expression of iNOS in the BFB and NO production increase during sleep deprivation [19].

Djungarian hamsters are nocturnal, seasonal mammals that adapt to seasonal changes in photoperiods and temperature with alterations to physiology, including sleep. The total sleep duration is similar between winter- and summer-adapted hamsters. In winter-adapted hamsters (short photoperiod) the distribution of sleep and wake is relatively even across the light and dark phases. Summer-adapted hamsters (long photoperiod) show more periods of wakefulness and fewer periods of SWS in the dark compared to the light phases [20]. They also exhibit greater power in slow wave activity during the light phase compared to the dark phase –an indication of greater drive to sleep that has been associated with adenosine receptor activation in mice [21]. Consequently, we hypothesized that adenosine levels may be greater in hamster BFB slices shortly after the dark/wake period in long-photoperiod (LP) adapted hamsters compared to the short photoperiod (SP) adapted hamsters. This has the experimental advantage that differences in sleep distribution can be observed in the absence of any additional handling of the hamsters and consequent potential stress.

The mechanisms of adenosine release in the BFB and sleep have largely been investigated *in vivo* by microdialysis. However, an *in vitro* model could be advantageous for the study of cellular and molecular mechanisms. To validate such a model, it is essential to illustrate that the mechanisms of sleep-dependent changes in extracellular adenosine in the BFB are still extant in *in vitro* preparations. Sleep-wake dependent changes in extracellular adenosine release have recently been demonstrated in hippocampal slices [22]. In this study we extend this approach to the BFB, which is causally linked to the homeostatic control of sleep. To demonstrate the generality of our results we have examined the relationship between adenosine release and sleep status in three species: rats, mice, and Djungarian hamsters. Our findings demonstrate that sleep-wake status has a biochemical memory that survives death and that the cellular mechanisms of adenosine production relevant to the control of sleep can be studied *in vitro*.

## Materials and Methods

### Slice Preparation

400 µm-thick coronal slices including the basal forebrain were obtained from several rodent species. These were 18–30-day-old, male, Sprague-Dawley rats; 2–3-month-old mice, C56BL/6 wild type littermates of transgenic dnSNARE mice; and 5–6-month-old Djungarian hamsters. All animal handling was carried out in strict accordance with the UK Animals (Scientific Procedures) Act 1986 (licence PPL 80/2493) with all effort taken to minimise suffering. Rats and mice were maintained on a 12 h/12 h light/dark cycle with lights on from 7 am to 7 pm. Unless otherwise stated, all animals were sacrificed approximately 2 hours into the light phase of the day (ZT 2). For some animals sacrificed at different points in the cycle, they were moved to a room on a different light cycle and given at least one week to acclimatise. Sleep deprivation was achieved by established principles of gentle handling [23], [24] or an 8229 Automated Sleep Deprivations System (Pinnacle Technologies Inc., USA) to minimise stress. Gentle handling was used for mice and 2 h sleep deprivation in rats, and the automated system for 6 h sleep deprivation in rats. Animals sleep deprived by automation were monitored every 45 mins for several minutes. Although EEG recordings were not used to assess sleep status of animals prior to sacrifice, the effects of adenosine release accumulate over hours of sleep status, which can be assumed to be consistent across multiple animals maintained in consistent conditions. These methods of sleep deprivation have well-established reliability and efficacy without the need for EEG recordings to confirm increased slow wave activity [22], [25]. For sleep recovery after deprivation, rats were marked, returned to a cage with their littermates and left for 24 h before sacrifice.

Animals were sacrificed by cervical dislocation, decapitated, and the brain was rapidly extracted and placed in a sub-4°C artificial cerebrospinal fluid solution (aCSF; see below for composition) with an extra 10 mM MgCl_2_ added to counteract excess NMDA receptor activation. Slices were cut on a Microm HM 650 V microslicer (Carl Zeiss, Welwyn Garden City, UK). Slices were then transferred to a holding chamber at room temperature in standard aCSF composed of (in mM): NaCl, 124; KCl, 3; CaCl_2_, 2; NaHCO_3_, 26, NaH_2_PO_4_, 1.2; MgSO_4_, 1; glucose, 10; equilibrated with 95%:5% O_2_:CO_2_ to pH 7.4. Slices were incubated for at least one hour prior to initial experiments.

### Recording and Analysis

For experiments, individual slices were placed on a nylon net, submerged in a recording chamber perfused with 32–33°C aCSF at a flow rate of 5–6 ml/min which was recycled, allowing sufficient run-out to waste during solution changes. Microelectrode biosensors (Sarissa Biomedical, Coventry, UK) were carefully placed in the slice visualised under an Olympus SZ60 stereozoom microscope (Japan). Sensors were inserted in pairs, one adenosine (ADO) or inosine (INO) sensitive and the other Null, in BFB and cortex at an angle of approximately 70° so that the active region was fully in the slice. Sensor pairs were placed as close together as possible consistent with the need to avoid crosstalk between biosensors and to minimize localised tissue damage. The areas of the BFB used were the horizontal arm of the diagonal band of Broca or substantia innominata. Initial insertion of bionsensors resulted in a transient purinergic response (20–40 minutes), which was allowed to decay before experiments commenced. After the experiments were completed, the biosensors were withdrawn from the slice and the response allowed to stabilise, to provide a baseline with which to measure the basal tone of the slice. They were then calibrated with aCSF containing 10 µM adenosine or inosine as appropriate, followed by aCSF with 10 µM serotonin to check that the sensor was adequately shielded and thus insensitive to other electro-active biological substrates. All drugs were bath applied by addition to the aCSF.

Biosensor signals were measured on a Duo-Stat ME200+ potentiostat (Sarissa Biomedical, Coventry, UK) and acquired on a DT3010 data acquisition board (Data Translation, Bietigheim-Bissingen, Germany). Offline data analysis was done with customised software written by N. Dale. Adenosine concentrations were measured at peak, and data are presented as mean ± standard error of the mean.

#### Biosensor characteristics

For a detailed explanation of the biosensors used, see Llaudet et al. [26]. Briefly, biosensors consisted of an etched, platinum wire 50 µm in diameter coated with a shielding layer against electro-active biological molecules (e.g. serotonin, dopamine, ascorbic acid) and a 500 µm-long active region of polymer matrix. In ADO sensors this matrix contained the enzymes adenosine deaminase, nucleoside phosphorylase and xanthine oxidase, breaking adenosine down to inosine, inosine to xanthine, and xanthine to uric acid and H_2_O_2_. ADO biosensors are also reactive to both inosine and hypoxanthine, which are endogenous to brain tissue. Consequently, all adenosine measurements are actually total purinergic responses, which is signified by the unit measurement ADO’. INO sensors, made as ADO except lacking adenosine deaminase, were used in some experiments to examine the time course of inosine responses. Null sensors contained no enzymes in the matrix, thus providing an internal control for non-specific electrical interferences. Consequently, all raw data traces show the subtraction current of the ADO or INO sensor minus the Null.

Biosensors could be expected to generate a current of 1–3 nA in response to 10 µM adenosine/inosine. Within the range of biological responses generated here, the relationship between purinergic concentration and current is linear [22], [26]. Evoked and tone adenosine concentrations were therefore calculated by the relationship of the experimental measurements to the 10 µM adenosine/inosine calibration at the end. All drugs were also tested against the biosensors with no slice present to ensure they did not intrinsically generate an electrical signal. Tone measurements were recorded as the difference in electrical signal recorded when the sensors were inserted in the slice and after removal before addition of the calibrating adenosine/inosine application.

#### Dependence of adenosine release on diurnal time

**Figure 1:**
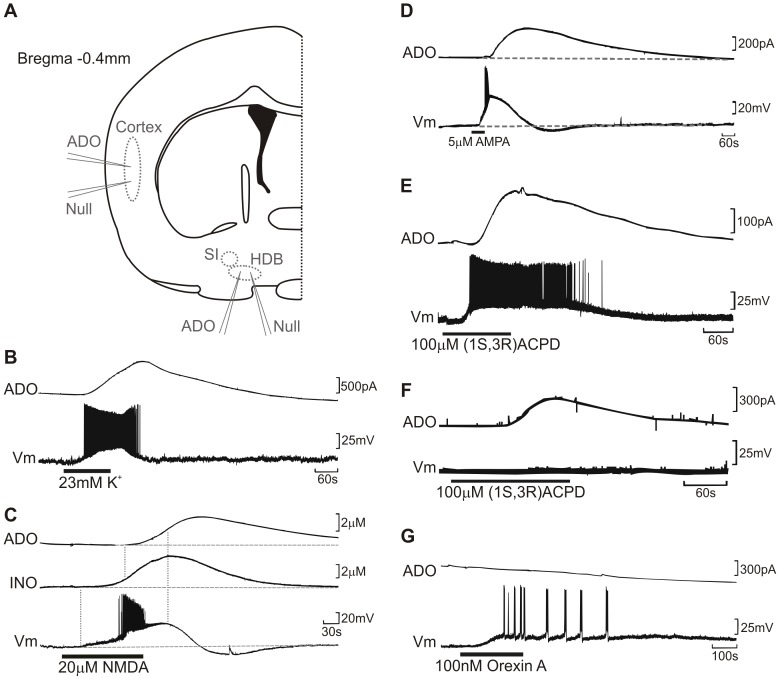
Release of adenosine by depolarisation and agonists. (a) Schematic diagram of a coronal slice indicating location of sensor placements. SI: substantia innominata; HDB: horizontal arm of the diagonal band. Adenosine release was measured as the peak ADO’ signal from BFB slices as measured by inserted biosensors combined with whole cell patch recordings of BFB neurons in current clamp. All panels show raw data from representative experiments. (b) High K^+^ aCSF evoked adenosine release and depolarisation of BFB neurons. (c) NMDA application also evoked neuronal depolarisation and firing accompanied by subsequent release of adenosine and inosine. (d) Adenosine released was evoked after depolarisation with AMPA (d), by (1R,3S) ACPD with or without accompanying depolarisation (e,f), and Orexin A did not induce ADO release despite causing depolarisation (g).

To investigate the dependence of adenosine release on the time of sacrifice of the animal we used 18–30-day-old rats. These were sacrificed at seven points in the 24 h cycle: ZT 2, ZT 6, ZT 9, ZT 12 (light), and ZT 14, ZT 17, ZT 20.5 (dark). Slices were prepared as above and allowed to rest 1 h prior to recordings. Biosensors placed in the BFB, and also the cortex as a control region not causally implicated in sleep as indicated in figure 1a. Adenosine release was evoked by a two-minute bath application of 5 µM AMPA in aCSF. After the response had returned to baseline, the biosensors were withdrawn from the slice and the current compared to the baseline to obtain the basal tone.

#### Dependence of adenosine release on sleep deprivation

To investigate the dependence of adenosine release on sleep deprivation prior to sacrifice, at the end of the sleep cycle, Sprague-Dawley rats were transferred to a new cage, and prevented from sleeping for 2 or 6 hours by established methods of gentle handling to minimize stress. They were then promptly sacrificed and slices prepared. As before, adenosine release was evoked by two-minute application of 5 µM AMPA and recorded by biosensors placed in the BFB and cortex. After the response had returned to baseline, the biosensors were withdrawn from the slice and the current compared to the baseline to obtain the basal tone.

#### Role of iNOS in triggering the enhanced adenosine release following sleep deprivation

2–3-month-old C57BL/6 mice were sleep deprived with gentle handling over a period of six hours after the end of the dark cycle, and coronal brain slices obtained as normal. Half the slices were incubated in aCSF containing 10 µM 1400 W for at least an hour prior to experimentation and maintained in 1400 W throughout the experiment. These mice were also compared to control mice sacrificed at a similar point in the diurnal cycle –6 h into the light cycle – that had not been sleep deprived.

#### Djungarian hamsters

5–6-month old Djungarian hamsters were separated into two groups and maintained in either a short (8 h light, 16 h dark) or long (16 h light, 8 h dark) photocycle, and left to acclimatize for 12 weeks. Their weight was measured every week to check that it was maintained in the long photoperiod group and diminished in the short photoperiod group. They were then sacrificed within two hours of the dark cycle ending. Biosensors were placed in both BFB and cortex. The testes were also dissected free and weighed to verify adaptation to photoperiod [27].

### Immunohistochemistry

Coronal slices containing BFB and cortex were prepared from 6 h sleep-deprived and non-sleep-deprived rats at a similar point in the diurnal cycle. Free-floating slices for iNOS immunofluorescence labeling were cut 150 µm thick, and slices for later cryosection and double immunofluorescence of iNOS and choline acetyltransferase (ChaT) were initially 300 µm thick. These slices were then incubated at room temperature in aCSF bubbled with 95%:5% O_2_:CO_2_ for 1 or 4 hours.

#### iNOSimmunfluorescence

Free-floating sections were fixed for 75 mins with 4% paraformaldehyde (PFA) at room temperature, followed by three rinses in TBS-Tween for 10 mins. For each immunohistochemical run, sections from Non-SD and SD brains were processed using the same reagents and conditions. After incubation in blocking solution (5% bovine serum albumin (BSA) in TBS-Tween) overnight at 4°C, sections were labeled with primary rabbit polyclonal antibody against inducible nitric oxide (iNOS; 1∶20, M-19; Santa Cruz Biotechnology) in 1% BSA in TBS-Tween overnight at 4°C. After thorough washing in Tris-buffered high salt saline (pH 8.6), sections were incubated for 1 hour with chick anti-rabbit AlexaFluor 488 nm secondary antibody (1∶1000; Invitrogen) at room temperature and mounted onto Superfrost plus slides and coverslipped with VECTASHIELD® mounting medium with DAPI (Vector Laboratories). Images of BFB and cortex were acquired using an SP2 confocal scanning microscope (Leica, Germany) under a 40x oil immersion lens and analyzed with Image J software.

#### Double immunofluorescence

For double immunofluorescence of iNOS and ChaT, slices were fixed for 1 h with 4% PFA in PBS at room temperature. Sections were washed in PBS and stored overnight at 4°C in 30% sucrose/PBS solution until sank. Brain slices were embedded in OCT and frozen sectioned using a sliding microtome into 17 µm coronal sections. Frozen sections were rinsed in PBS, washed twice with TBS and permeabilized with TBS-Tween for 15 mins at room temperature. The sections were pre-incubated for 1 hour at room temperature in blocking solution and then double labeled with a rabbit anti-iNOS polyclonal antibody (1∶20, M-19; Santa Cruz Biotechnology) and a sheep anti-ChAT polyclonal antibody (1∶20, Ab18207; Abcam) in 1% BSA in TBS-Tween overnight at 4°C. The immunoreactivities to iNOS and ChaT were visualized using a chicken anti-rabbit AlexaFluor 488 nm- and a donkey anti-sheep AlexaFluor 594 nm-conjugated secondary antibodies. Immunohistochemical studies for all sections were stained with DAPI (1∶1000; Invitrogen) and mounted in Mowiol 4–88 (Sigma). Secondary antibody in 1% BSA in TBS-Tween without primary antibody was used as a negative control to detect the specificity of immunofluorescence. Images were acquired as above.

### Drugs

All drugs were made up as stock solutions in distilled water, frozen for storage, and diluted 1∶1000 in aCSF to their final bath concentration. 2-amino-3-(5-methyl-3-oxo-1,2- oxazol-4-yl)propanoic acid (AMPA) and N-methyl-D-aspartic acid (NMDA) were supplied by Ascent Scientific (Bristol, UK). 1400 W and erythro*-*9-(2-Hydroxy-3-nonyl)adenine (EHNA) were supplied by Tocris-Cookson (Bristol, UK). All other drugs were were supplied by Sigma-Aldrich (Dorset, UK). Concentrations used were those established as efficacious in prior studies, e.g. histamine [28], orexin A [29], neurotensin [30]. In the case of AMPA and NMDA, a dose close to the EC50 to reduce concerns regarding excitotoxicity.

### Statistical Analysis

Observation of histograms suggested non-normal distribution, although some data sets were normally distributed according to Shapiro-Wilk test. Consequently the non-parametric Mann-Whitney U test or Wilcoxon signed-rank test (both one-tailed, on the basis that the manipulations we were performing would be expected to increase adenosine release) were used. The False Discovery Rate procedure was used [31] to minimise the possibility of false rejections of the null hypothesis during multiple comparisons. We used an upper limit for the false discovery rate of 0.05. Statistical analysis was carried out on PASW Statistics 18 software (IBM, USA). The n values refer to the number of slices used, with one recording per slice and 2–4 slices per animal.

## Results

Extracellular adenosine levels *in vivo* are likely to depend on both the extent of neuronal activity [32]–[36] and on the rate of basal release. In brain slices the basal mechanisms of release are likely to be preserved but the levels of spontaneous neuronal activity are much less than *in vivo*. We therefore examined extracellular adenosine in two ways: the basal adenosine tone, and during depolarizing stimuli (to probe potential activity dependence of adenosine release).

### Basal Tone of Adenosine

We first examined the basal tone of purines in the BFB (n = 12). The ADO’ basal tone was 0.56±0.15 µM and INO sensor basal tone was 0.28±0.04 µM, suggesting that 49% of the ADO’ tone was adenosine. We verified this measurement by an alternative method: use of an adenosine deaminase inhibitor EHNA, to inhibit adenosine deaminase within the biosensor and thus selectively block the adenosine component of the biosensor signal [34]. Application of 20 µM EHNA decreased the tone by 0.22±0.10 µM, indicating that by this method 39% of the signal was adenosine.

### Depolarizing Stimuli Evoke Adenosine Release

#### High K^+^

*In vivo*, BFB extracellular adenosine levels in rats are observed to increase with increased wakefulness and decrease with increased sleep, and increase considerably more during sleep deprivation. This is consistent with evidence of adenosine release due to neuronal activity [17], [37]–[39], as BFB cellular activity increases during periods of wakefulness and is considerably lower during NREM sleep according to juxtacellular recordings [40], [41] and c-fos activity [42]. Whole cell patch clamp recordings were made from BFB neurons whilst extracellular adenosine and inosine concentrations were concurrently recorded with biosensors. Depolarization was evoked by applying a high K^+^ (23 mM) aCSF (figure 1b). High K^+^ caused depolarization of neurons coupled with intense action potential firing, and then after a brief delay adenosine and inosine release.

#### Glutamatergic agonists

We next tested the ability of more selective depolarizing stimuli to evoke adenosine release in basal forebrain slices. We examined ionotropic glutamate receptors, which are are the most common source of excitatory inputs in the brain. Furthermore, *in vivo* experiments have shown BFB adenosine release by microdialysis application of AMPA and NMDA [43].

Simultaneous whole cell patch clamp and biosensor recordings in BFB showed that 20 µM NMDA applied to the bath caused transient firing followed shortly later by adenosine release, 1.16±0.26 µM ADO’, 144±38 s to peak, n = 6 (figure 1c). The role of AMPA receptors was then examined with application of 5 µM AMPA to the aCSF (figure 1d). This caused rapid depolarization of neurons and after a delay by the release of adenosine (1.21±0.34 µM, n = 17). The percentage of the ADO’ signal that was adenosine also was examined with ADO and INO sensors placed in the BFB. Upon addition of 5 µM AMPA the mean peak ADO’ concentration (ADO-Null) was 1.1±0.5 µM, with a mean latency of 211±11 s from initial AMPA application, n = 9. At the ADO’ peak, the INO response (INO-Null) was 0.7±0.3 µM, suggesting 40% of the AMPA-evoked ADO’ signal is adenosine at peak, and 60% inosine/hypoxanthine (data not shown).

Metabotropic glutamate receptors (mGluRs) have also been reported present in BFB neurons and glia [44]–[46], and so mGluRs were also tested to see whether they evoked adenosine release. The group I mGluR agonist (1S,3R) ACPD at 50–100 µM reliably evoked adenosine release, although this was not reliably accompanied by neuronal depolarization (figures 1e,f). Peak ADO’ concentration was 2.84±1.12 µM. Neither L-AP4 nor (2R,4R) APDC caused adenosine release, suggesting that neither group II nor group III mGluRs are involved.

#### Other depolarizing stimuli

BFB neurons are known to be receptive to several other neurotransmitters which are capable of depolarizing cells. Orexin, acetylcholine (ACh), neurotensin and histamine are all transmitters associated with the ascending arousal system. However, application of 100 nM Orexin A failed to generate adenosine release despite causing depolarizing and action potentials in BFB neurons (figure 1g), which was also observed with 200 nM neurotensin, 100 µM histamine and 100 µM ACh.

Therefore in the BFB, although widespread depolarization with high K^+^ or glutamatergic agonists is sufficient to generate adenosine release, not all depolarizing stimuli can release adenosine. There may be a discrete population of BFB cells – neurons or astrocytes – with a specific receptor phenotype that are responsible for adenosine release. These cells possess ionotropic glutamate receptors and group I mGluRs, but have few or lack orexin, histamine and neurotensin receptors.

### Ca^2+^-independence of Evoked Adenosine Release

**Figure 2:**
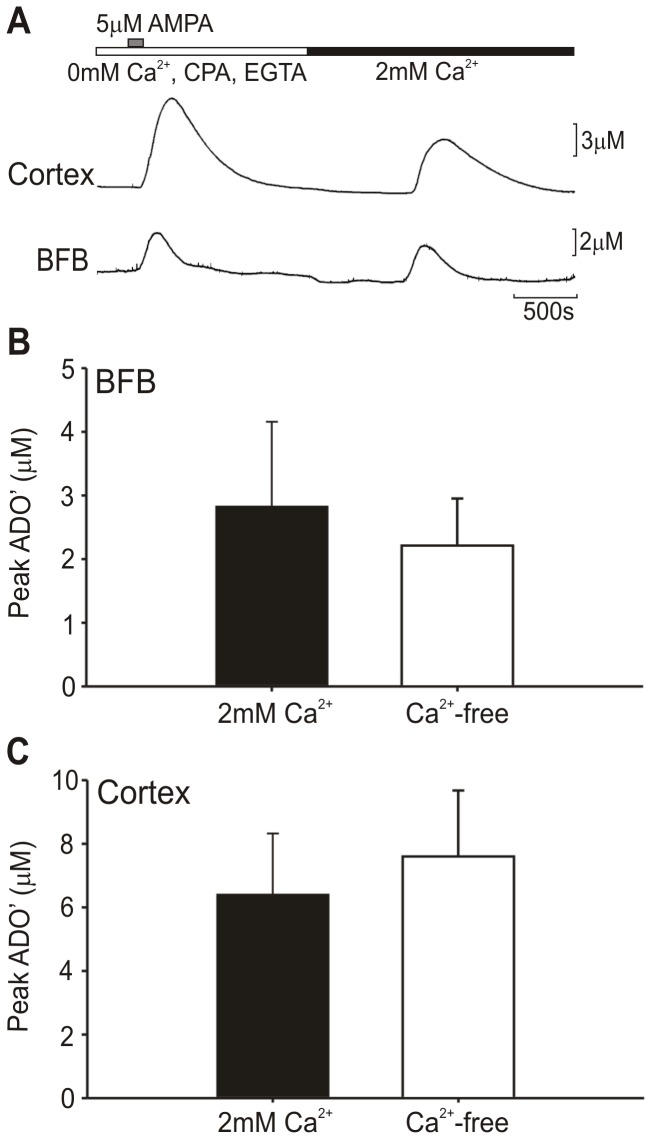
Adenosine release occurs independently of calcium. Adenosine release was evoked by 5 µM AMPA in Ca^2+^-free aCSF (0 mM Ca^2+^ and 1 mM EGTA) with 20 µM CPA added to drain internal calcium stores, and then again after following wash in of normal 2 mM Ca^2+^ aCSF. (a) Raw data traces from a representative experiment in cortex (top) and BFB (bottom). Adenosine responses were not significantly different in Ca^2+^ -free conditions in either BFB (b) or cortex (c) by the Mann-Whitney U-test, n = 6.

As depolarizing stimuli could indirectly cause release of adenosine through the actions of interposed transmitters, we tested the Ca^2+^ dependence of adenosine release. ADO and Null biosensors were placed in the BFB and cortex. CaCl_2_ was removed from the aCSF and replaced with 2 mM MgCl_2_ and 1 mM EGTA. Furthermore, 20 µM cyclopiazonic acid (CPA), an inhibitor of Ca^2+^-ATPase, was included in order to drain internal Ca^2+^ stores. 5 µM AMPA was used to evoke adenosine release, and then normal aCSF washed back in and AMPA applied again (figure 2a). Adenosine release was not significantly different in presence or absence of Ca^2+^ in either the BFB (Ca^2+^-free: 2.21±0.74 µM; 2 mM Ca^2+^: 2.82±1.33 µM; figure 2b) or cortex (Ca^2+^-free: 7.60±2.08 µM; 2 mM Ca^2+^: 6.40±1.92 µM; figure 2c) according to the Wilcoxon signed-rank test, n = 7. These results indicate that the mechanism of AMPA-evoked adenosine release in this *in vitro* preparation is independent of both intra- and extracellular Ca^2+^, indicating that it may not be mediated by vesicular exocytosis.

### Diurnal Rhythms of Adenosine Release and Tone

Microdialysis experiments *in vivo* have demonstrated basal adenosine levels throughout the brain exhibit a diurnal rhythm, increasing during wakefulness and decreasing during sleep [7], [8], [10]. Although this appears to be true throughout the brain, it is particularly prominent in the basal forebrain. We therefore investigated whether adenosine release *in vitro* exhibited a prior dependence on the time of sacrifice of the animal relative to the light-dark cycle.

#### Measurements from rat slices

**Figure 3:**
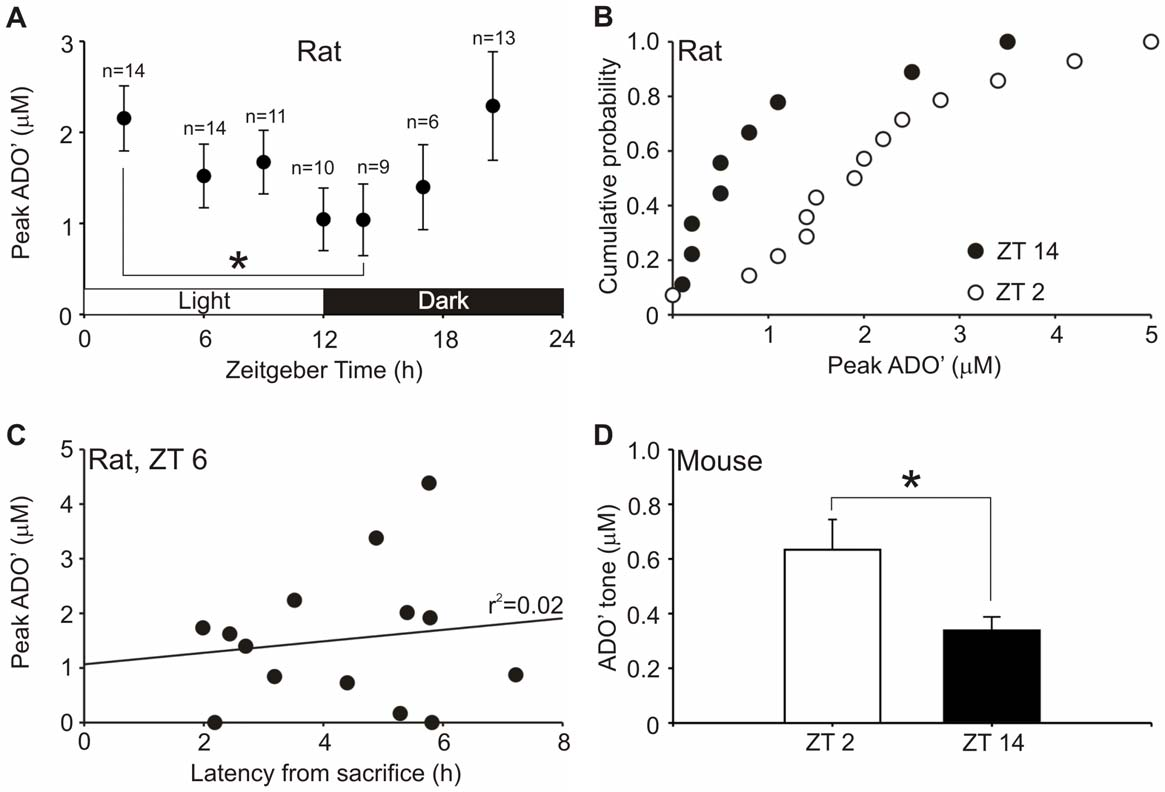
AMPA-evoked adenosine release varies with diurnal cycle in the basal forebrain. Animals were sacrificed at various times in the diurnal cycle and adenosine release from slices evoked by 5 µM AMPA. (a) In rat BFB, adenosine release varied with time of sacrifice, n values for each point as indicated in the figure. (b) Cumulative probability distributions of individual peak ADO’ responses at ZT 2 and 14 in rats. (c) ADO’ responses were not affected by the time slices were left to incubate following sacrifice and preparation, illustrated for slices used at ZT 6. (d) In mice, greater BFB ADO’ tone was also observed after wake periods as indicated by recordings at ZT 2 and ZT 14. Asterisks indicate significant differences, p<0.05, Mann-Whitney U test.

AMPA-evoked adenosine release showed systematic variation with time of sacrifice. The release increased the farther into the dark cycle the rat was sacrificed, and conversely decreased as sacrifice was performed later into the light cycle (figure 3a). AMPA evoked release at ZT 2 (2.2±0.4 µM; n = 14) was significantly higher (p<0.05, Mann-Whitney U test,) than at the opposite point in the wake/sleep cycle at ZT 14 (1.0±0.4 µM; n = 9; figure 3b).

No significant cycle was observed in the basal tone of rat slices, although this is most likely due to natural variability given the very small adenosine concentrations in tone, range 0.2–0.6 µM in rats. Furthermore, no adenosine cycle was observed from the cortex either from AMPA-evoked release or basal tone. AMPA-evoked cortical responses were, however, 5–10 times greater in peak magnitude than BFB responses (range 5.5–12.4 µM), and of greater duration.

Adenosine release was similar in the horizontal arm of the diagonal band of Broca and the substantia innominata (SI). Nor were responses observed to vary according to the latency between sacrifice and start of experiment, indicating that length of incubation did not appear to alter adenosine release, further suggesting that adenosine responses are representative of time of sacrifice. This is illustrated for ZT 6 (figure 3c).

Our results suggest that in slices of the BFB, aspects of adenosine release are sensitive to the diurnal cycle. These altered concentrations of adenosine cannot result from simple accumulation of adenosine in the extracellular environment while *in vivo*, as this would be washed out of the slice during incubation and perfusion. Instead the changes in extracellular adenosine must in some way reflect changes in the molecular, biochemical and cellular properties of slices derived at different times of sacrifice such that alters the balance between release and uptake from cells.

#### Measurement from mouse slices

Similar experiments were performed to see if the same differences could be observed in mice, using only two times of sacrifice separated by 12 h, 2 h into the light cycle (ZT 2) and 2 h into the dark cycle (ZT 14). The AMPA-evoked release from BFB in mouse slices was not significantly different between ZT 2 (0.12±0.04 µM, n = 21) and ZT 14 (0.10±0.05 µM, n = 17). However, basal tone was significantly greater at ZT 2 (0.63±0.11 µM) than ZT 14 (0.34±0.05 µM) according to the Mann-Whitney U-test, p<0.05 (figure 3d). No difference was observed between AMPA-evoked and basal tone adenosine in the cortex. These data indicate that a difference in adenosine release according to diurnal rhythm can also be observed in mice and accords with the data of Schmitt et al. [23] documenting adenosine release in the hippocampus.

### Effects of Prolonged Wakefulness

Any mechanism involved in the control of sleep should exhibit sensitivity to prolonged wakefulness. We have therefore examined this in three rodent models: the rat, as this has extensively been used in prior *in vivo* studies; the mouse, as this opens the possibility of using genetic modifications to examine mechanism; and the Djungarian hamster which under goes natural variation in its sleep patterns when adapted to different photoperiods.

#### Rats

**Figure 4:**
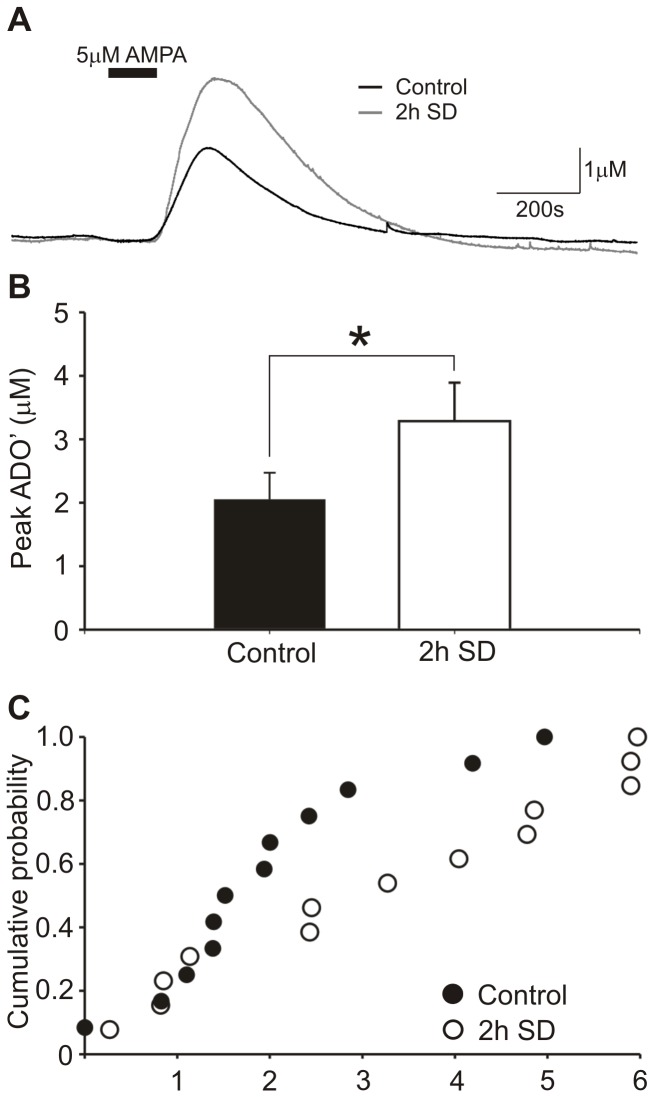
2h sleep deprivation causes an increase in basal forebrain adenosine release in rats. Adenosine release evoked by AMPA in slices from 2 h SD rats was compared to control rats sacrificed at the same point in the diurnal cycle without sleep deprivation. (a) Raw data traces of representative experiments of sleep deprived and control rats adjusted to the adenosine calibration. (b) Slices from 2 h SD rats (n = 13) had greater ADO’ responses than controls (n = 14) with the asterisk indicating significance (Mann-Whitney test, p<0.05). (c) The cumulative probability graph illustrates the distribution of individual ADO’ responses for control (black fill) and 2 h SD (white fill).

Prolonged wakefulness arising from sleep deprivation is also known to cause a considerable increase in extracellular adenosine concentrations *in vivo* [47]. We found that ADO’ responses to AMPA in 2 h sleep deprived (SD) rats were 160.0% that of control animals sacrificed at a similar point in the diurnal cycle (control: 2.0±0.4 µM, n = 14; SD: 3.3±0.6 µM, n = 13; Mann-Whitney U test, p<0.05; figure 4). Tone measurements of BFB were higher in 2 h SD (0.50±0.08 µM) rats than control (0.33±0.08 µM), but not significantly so (p = 0.14, Mann-Whitney U test). However, in the cortex there was no observed statistical difference in AMPA-evoked adenosine release (control 9.7±1.6 µM; SD 11.4±1.3 µM) or tone (control 0.88±0.12 µM; SD 0.76±0.23 µM).

Recent *in vivo* data has suggested that adenosine concentrations following 6 hours of sleep deprivation depend on nitric oxide (NO) and induction of iNOS [18], [19]. We therefore examined whether the enhanced release of adenosine in slices following sleep deprivation also depended on activation of iNOS. Peak ADO’ in slices from 2 h SD rats incubated with 1400 W (3.5±0.7 µM, n = 6) was not significantly different from those without 1400 W.

Although induction of iNOS, production of NO (as indirectly measured by downstream metabolites NO_2_^−^ and NO_3_^−^) and ADO release begins under 1 h into sleep deprivation [18], at 2 h all three are sub-maximal [19]. Consequently, we examined both adenosine release and the basal tone after a longer period of sleep deprivation to ensure adequate induction of iNOS.

**Figure 5:**
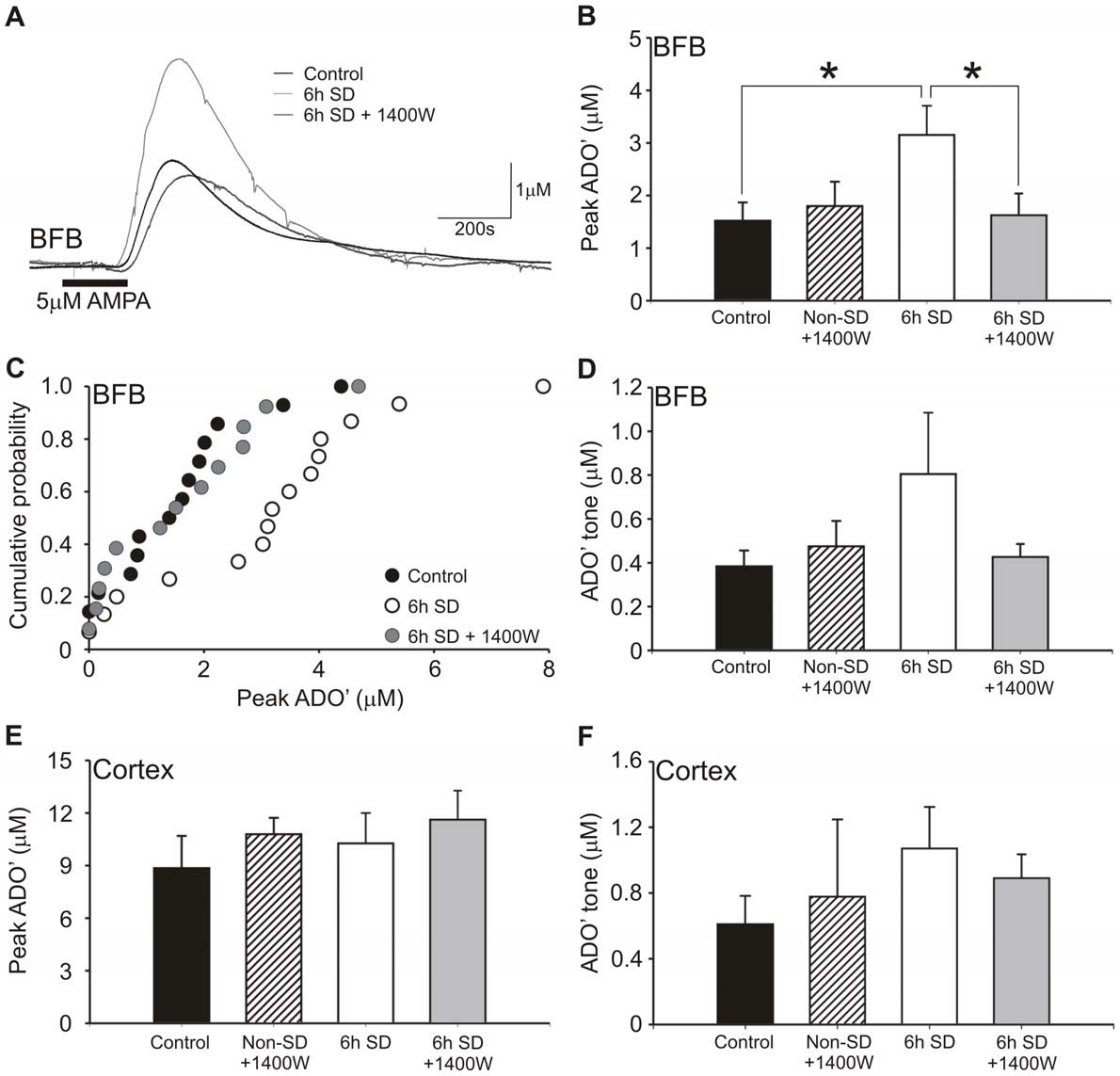
6h sleep deprivation causes an iNOS-dependent increase in adenosine release in rats. Adenosine release was compared between 6 h sleep deprived and non-sleep deprived rats sacrificed at the same point in the diurnal cycle in the presence and absence of 1400 W. (a) Raw data from representative experiments of non-SD controls (black), 6 h SD (light grey) and 6 h SD +1400 W (dark grey) experiments, normalized to 10 µM ADO calibration. (b) In the BFB, slices from 6 h SD (n = 14) showed greater ADO’ release than those from control rats (n = 13) and 6 h SD rats +1400 W (n = 14), and non-sleep deprived rats +1400 W (n = 8) rats were not significantly different from controls. (c) Cumulative probability distribution of individual peak ADO’ responses for control (black fill), 6 h SD (white fill) and 6 h SD +1400 W (grey fill) in BFB. (d) No conventional significant difference (6 h SD v. control p = 0.09) was observed in basal adenosine tone for the same experiments in BFB. In the cortex, there were no significant differences in either the AMPA-evoked release (e) or basal tone (f). Asterisks indicate statistical significance (Mann Whitney U test, p<0.05).

AMPA-evoked release in the basal forebrain after 6 h sleep deprivation in rats was 207% that of control rats sacrificed at a similar point in the diurnal cycle (control: 1.5±0.3 µM, n = 13; 6 h SD: 3.3±0.6 µM, n = 14) and also significantly greater than slices from 6 h SD rats incubated with 10 µM 1400 W (1.6±0.4 µM, n = 14, Mann-Whitney U-test, p<0.05; figures 5a–c). Between all three comparisons, significance was maintained by false discovery rate analysis. Although the basal ADO’ tone was greater after 6 h SD (0.80±0.28 µM) than control (0.38±0.07 µM) and 6 h SD +1400 W (0.42±0.06 µM), this failed to achieve conventional statistical significance (p = 0.09 control v. SD; p = 0.14 SD v. 1400 W, Mann-Whitney U test; figure 5d).

Slices from control rats 6 h into the light cycle were also incubated with 1400 W to investigate whether it had any effect on adenosine release without sleep deprivation. For slices prepared from non-sleep deprived rats, 1400 W had no effect on the AMPA-evoked release in either basal forebrain or cortex (BFB: 1.8±0.5 µM; Ctx: 10.8±0.9 µM), and was not significantly different from controls in the absence of 1400 W. This was also true for the effect of 1400 W on the ADO tone in non-sleep-deprived slices (BFB: 0.52±0.15 µM; Ctx: 0.78±0.48 µM). Thus the actions of iNOS are specific to the sleep deprived state.

In the cortex, AMPA-evoked adenosine release was 8.9±1.8 µM (n = 10) in slices from control rats, 10.3±1.7 µM (n = 13) from 6h SD rats and 11.6±1.7 µM (n = 11) for 6 h SD +1400 W (figure 5e). Basal tone was 0.61±0.17 µM for control, 1.07±0.25 µM for 6 h SD, and 0.89±0.15 µM for SD +1400 W (figure 5f). None of these were significant by the Mann-Whitney U test (AMPA-evoked: p = 0.29 control v. 6 h SD; p = 0.22; 6 h SD v. 6 h SD +1400 W; for tone: p = 0.13 control v. 6 h SD; p = 0.35 6 h SD v. 6 h SD +1400 W). As with BFB, slices from non-sleep deprived rats incubated with 1400 W (n = 7) were similar to controls in AMPA-evoked release and basal tone.

Some rats subjected to 6 h SD were also allowed 24 h recovery sleep before sacrifice. For these experiments (n = 15) in the basal forebrain AMPA-evoked ADO release was 1.8±0.5 µM and basal tone 0.36±0.05 µM. In the cortex, AMPA-evoked ADO release was 9.4±1.8 µM and basal tone 0.71±0.27 µM. These results were not significantly different from non-sleep deprived controls, and only AMPA-evoked release was significantly lower than 6 h SD without recovery sleep. (Mann-Whitney U-test, p<0.05).

#### Immunocytochemical localization of iNOS following sleep deprivation

**Figure 6:**
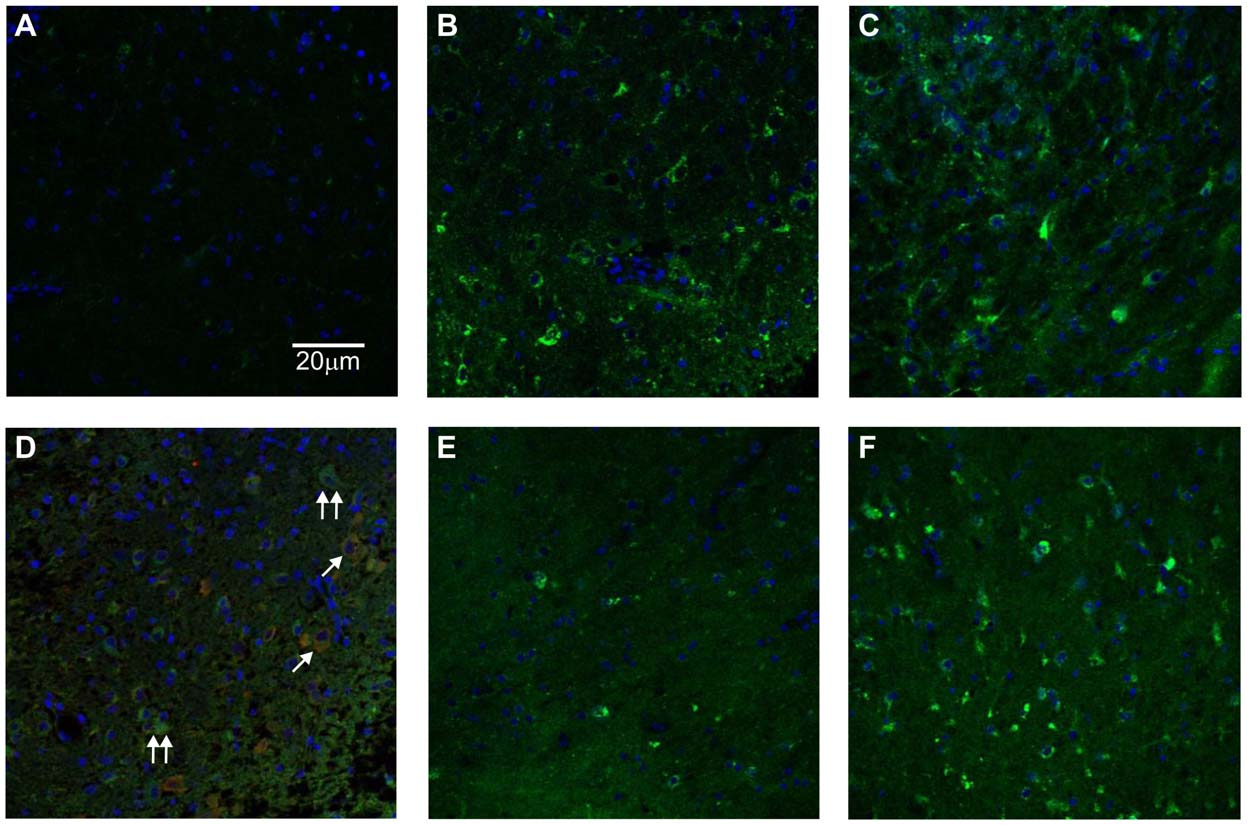
6h sleep deprivation causes an increase in iNOS expression in BFB and cortex. iNOS Immunofluorescence (green) was largely absent in BFB slices from rats not sleep deprived after 1 h post-sacrifice incubation in aCSF (a), but strong after 6 h SD with 1 h (b) and 4 h (c) post-sacrifice incubation. The blue immunfluorescence is DAPI, showing nuclei. Double immunfluorescence staining for iNOS and ChaT (red) in BFB for a 6 h SD rat after 1 h incubation is shown in (d), single arrows indicate examples of somata with colocalised ChaT and iNOS, double arrows somata with iNOS but no ChaT. In the cortex, iNOS immunfluorescence after 4 h incubation was present in non-SD rats (e), but less strong than those after 6 h SD (f).

To check that iNOS was present in our slices, we carried out immunfluorescence staining for iNOS. Rats that were sleep deprived for 6 h were compared to non-sleep deprived rats at a similar point in the diurnal cycle. After sectioning, slices were incubated in aCSF as normal for 1 h, then free-floating slices fixed and stained. Non-sleep-deprived rats had very little iNOS immunfluorescence in the BFB (figure 6a) in contrast to considerable fluorescence in slices from those that were sleep deprived (figure 6b). To see whether iNOS expression might decay during prolonged incubation of slices after sacrifice, we also processed slices that had been incubated for 4 h after sacrifice, and again observed considerable iNOS immunofluorescence (figure 6c). By contrast after 4 h incubation following sacrifice, there was still insignificant iNOS present in slices from non-sleep deprived rats. Joint immunofluorescence for iNOS and ChaT indicated that iNOS was present in ChaT positive cells (figure 6d), but not exclusive to them, suggesting that non-cholinergic cells in the BFB are also involved in the increase of NO production. Finally, the cortex was also examined with iNOS in free-floating slices. As with the BFB, iNOS immunofluorescence was considerably greater in sleep-deprived animals, although there was some observed in non-sleep deprived too (figures 6e, f).

#### Mice

**Figure 7:**
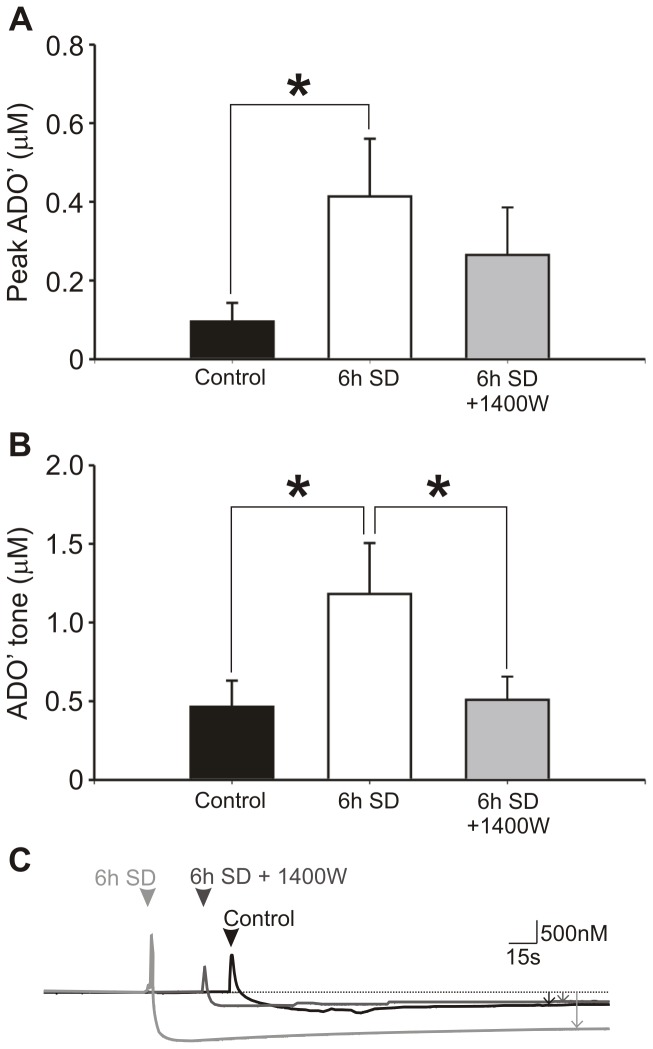
6h sleep deprivation causes iNOS-dependent increases in basal forebrain adenosine release in mice. Comparison of adenosine release and tone following 6 h sleep deprivation in mice either with or without 1400 W. (a) AMPA-evoked release was significantly higher in slices after 6 h SD (n = 15) than control (n = 10), but was not significantly greater than 6 h SD incubated with 1400 W. (b) Basal tone however was significantly greater in 6 h SD mice than when incubated with 1400 W and also controls (b). Asterisks indicate significant difference (Mann-Whitney test, p<0.05). (c) Raw data traces from representative experiments for control (black) 6 h SD (light grey) and 6 h SD +1400 W (dark grey) normalised to 10 µM ADO’ calibration for tone measurements are shown (c). Bold arrows indicate the point of sensor removal from the slice causing artefacts, and those on the right tone measured by difference before and after removal.

We next examined the effect of sleep deprivation effects in slices derived from mice. Again, sleep deprived animals were matched against control animals sacrificed at a similar point in the diurnal cycle without sleep deprivation. Following 6 h SD in mice, AMPA-evoked responses were higher (0.46±0.15 µM, n = 15) than in control (0.10±0.05 µM, n = 10), significantly so by Mann-Whitney U-test, p<0.05 but not by false discovery rate analysis applied to control, 6 h SD and 1400 W (figure 7a). In 6 h SD mouse slices treated with 1400 W, AMPA-evoked responses were reduced compared to untreated 6h SD slices but this did not achieve statistical significance (0.26±0.12 µM, n = 15).

The basal adenosine tone in control mice was 0.46±0.17 µM and significantly higher in 6 h SD mice at 1.18±0.32 µM (Mann-Whitney U test, p<0.05, figures 7b,c). In 6 h SD +1400 W mice the tone was similar to controls and significantly less than 6 h SD without 1400 W (0.51±0.15 µM). Significant differences between groups were sustained by false discovery rate analysis. In the cortex, however, no significant differences were observed between controls, 6 h SD and 6 h SD +1400 W in either AMPA-evoked release or basal tone. Our results suggest that there are significant differences between rats and mice in the magnitude of activity-dependent and basal adenosine release and that mice may be advantageous for measurement of adenosine tones whereas rats by contrast are more advantageous for examining AMPA-evoked adenosine release.

#### Hamsters

**Figure 8:**
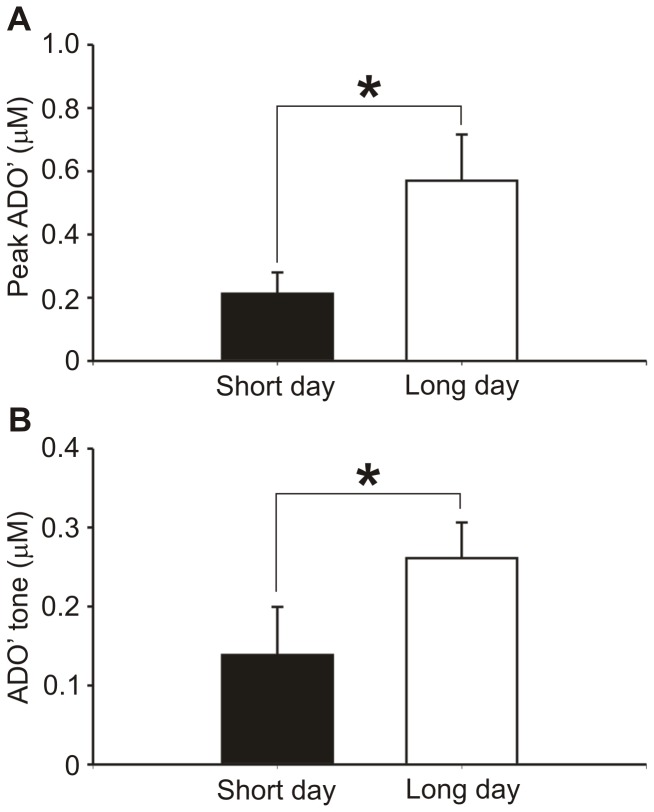
Differential adenosine release in basal forebrain due to seasonal sleep patterns of Djungarian hamsters. Slices from Djungarian hamsters kept in either long photoperiod (n = 15) or short (n = 14) light cycles were sacrificed two hours after the end of the dark phase were used. Both AMPA-evoked ADO’ release (a) and basal ADO’ tone (b) in the BFB were significantly greater in long day cycle hamsters (Mann-Whitney test, p<0.05).

Both a greater adenosine release (evoked by 5 µM AMPA) and resting tone was observed in LP compared to SP hamsters (LP hamsters AMPA: 0.18±0.04 µM; tone 0.57±0.15 µM; n = 15; SP hamsters AMPA: 0.06±0.04 µM; tone 0.21±0.07 µM; n = 14). These differences were statistically significant, p<0.05, Mann-Whitney U-test (figure 8). This provides evidence from a third rodent model that adenosine concentrations appear to differ by sleep status *in vitro*. Furthermore, it validates previous data indicating the different sleep patterns in hamsters according to seasonal variation.

## Discussion

Our results suggest that both activity dependent and basal adenosine release in the BFB *in vitro* exhibit sensitivity to the diurnal time of sacrifice and any sleep deprivation prior to sacrifice. The enhanced adenosine release or tone observed *in vitro* following sleep deprivation exhibited dependence on iNOS. This suggests that the same fundamental mechanism (dependence on iNOS and NO signaling) controls the availability of adenosine for both activity-dependent and basal release in this brain area. Our *in vitro* model therefore recapitulates key aspects of the homeostatic control of sleep that have previously been described from *in vivo* studies in the BFB. Our work demonstrates that the sleep status of an animal is encoded in a biochemical state or “memory” that can survive death, and that this is likely to be the induction of iNOS.

### 

#### Characteristics of adenosine release

Activity dependent adenosine release has been described in several different contexts (reviewed by Wall & Dale [35]). Some of this arises from previously released ATP that is broken down into adenosine in the extracellular space. However in the best-studied example, adenosine can be released directly from cerebellar parallel fibres via exocytosis [32], [34], [36].

Increases in extracellular adenosine concentrations are also associated with metabolic stress [37], [48], [49]. Adenosine concentrations also increase during seizure activity as a neuroprotective mechanism [50], [51]. Here it is likely that adenosine first accumulates intracellularly and transported across the plasma membrane via the equilibrative nucleotide transporters. This mechanism of direct activity dependent adenosine release has also recently been proposed to result from the metabolic load of neuronal firing so that adenosine can act as an autocrine modulator in the brain [33].

The BFB is a heterogeneous region containing cholinergic cells, GABAergic and glutamatergic cells, which receives afferents from wake-promoting areas of the brainstem. Orexin inputs are known to derive from the hypothalamus and innervate cholinergic centres of the BFB [52], with release associated with waking and REM sleep [53]. Neurotensin activates and promotes bursting behaviour in BFB cholinergic cells [54]. Histaminergic cells in the tuberomammillary nucleus also innervate BFB cholinergic neurons, and are most active in vivo most during wakefulness [55]. The failure of non-glutamatergic neurotransmitters to stimulate adenosine release in slices poses several possibilities: 1) they innervate insufficient neurons or otherwise generate insufficient neuronal activation, 2) adenosine release is primarily from a neuronal subpopulation not responsive to them, 3) they generate very specific cellular responses that may not include adenosine release.

A possibility for adenosine release is that it is astrocytic rather than neuronal. Although astrocytes are not electrically excitable, they do respond to neuronal activity, with intracellular calcium concentration increases [56] and releasing neurotransmitters such as glutamate [57], ATP [58], and D-serine [59]. However, the calcium independence of adenosine release observed here makes it unlikely that it primarily derives from astrocytic, exocyotic release of ATP. Astrocytes are theorized to act as a homeostatic regulator of brain function, including monitoring and consequently modulating synaptic activity, a role in at least partly mediated via purinergic signaling [60]. Astrocytes possess a wide range of glutamate receptors [61]–[63] and furthermore are already known to release ATP that is then converted to adenosine [64], [65]. It is unknown whether astrocytes in the BFB respond to orexin, histamine and neurotensin, although it should be expected that some indirect response should be expected through activated cholinergic cells.

#### Increased adenosine from prolonged wakefulness

*In vivo* microdialysis studies report BFB adenosine concentrations of 12–70 nM adenosine in rats and cats [9], [47], [66], [67]. However, microdialysis probes are estimated to recover only 10–20% of the adenosine [66], suggesting estimates in the low hundreds nanomolar range. As adenosine release is activity dependent and given the increased metabolic rate of the brain during wakefulness [68], [69], elevation of adenosine concentrations have been observed to follow a diurnal rhythm, increasing during the wake phase and declining during sleep [9], [70]. Sleep deprivation for 6 h in cats induced a 40% increase in BFB adenosine levels [7], [8].

In our slices, basal tone should reflect the underlying activity of cells in a relatively quiescent state, whereas depolarization or glutamatergic agonists would mimic the role of neuronal activity in adenosine release by causing generalized cellular activity. Consequently both could reflect changes in the intrinsic mechanisms of adenosine production and release. Interestingly, tone measurements did not reveal significant differences in rats whereas AMPA-evoked release did, with the reverse true in mice. This is possibly due to low basal ADO’ concentrations and the variability of responses between slices, but may represent a species difference.

Basal extracellular adenosine tones in slices indicate that there is a constant background degree of adenosine production and release still evident. Here the tone was measured in the region of 200–500 nM ADO’ throughout the diurnal cycle, of which 40–50% was adenosine. This is similar to that observed *in vivo*. However as numerous axonal connections are necessarily severed during slice preparation there is likely to be less cellular activity in slices than *in vivo*.

This study demonstrates that our *in vitro* model substantially replicates data acquired *in vivo*. AMPA-evoked adenosine responses varied according to both the diurnal cycle and due to sleep deprivation, and tone measurements also showed increases due to sleep deprivation.

#### The role of iNOS in sleep deprivation

The rise in BFB adenosine concentrations due to sleep deprivation is dependent on the neuromodulator NO produced via iNOS. Concentrations of nitrate and nitrite – indirect measurements for NO – doubled in the basal forebrain throughout sleep deprivation [17], [18]. Inhibiting iNOS with a specific inhibitor or non-specific NOS inhibitor, or scavenging NO with cPTIO all prevented adenosine increases from sleep deprivation, indicating adenosine production enhancement is downstream of NO release. More recently, further studies have indicated an increase in the expression of iNOS mRNA and protein in both the BFB and frontal cortex, although the latter over 3 h later, with an increase in NO-derivatives preceding adenosine increase [19].

Importantly, as inhibition of iNOS with 1400 W *in vivo* inhibited SD-derived increases in adenosine concentrations [18], so 1400 W also prevented the increase in adenosine concentrations recorded *in vitro* following SD, but otherwise had no effect on adenosine release or tone under control conditions. The diurnal variation of adenosine release must therefore occur through a mechanism independent of iNOS. This is a powerful indication that our model represents mechanistic aspects of physiological conditions, and crucially also suggests that the mechanisms of increased adenosine release are retained even after sacrifice, so must be to some extent ‘hard-wired’ into cellular activity.

#### Brain region specificity

Our results also indicated the consistent lack of any sleep-status-dependent difference in adenosine concentrations in the cortex, either in basal tone or AMPA-evoked release. This reinforces the possibility that alterations of adenosine concentrations due to sleep status are region-specific in the brain. *In vivo*, sleep deprivation leads to a slower rise and smaller increase in adenosine in the cortex compared to BFB across the first 5 h [19]. Earlier studies suggested no significant increase in other brain areas associated with sleep such as the thalamus, dorsal raphe nucleus, pedunculopontine tegmental nucleus and preoptic hypothalamus [7], [8]. After 3 h recovery sleep, ADO concentrations were only elevated in the BFB. Perfusion of nitrobenzylthioinosine, a blocker of equilibrative nucleoside transporters, caused an increase in extracellular BFB adenosine and subsequent increased slow wave sleep.

Interestingly in the hippocampus, and using similar methods to those reported here, Schmitt et al. [22] have obtained clear evidence for a diurnal variation of adenosine tone and its sensitivity to SD. In this case the adenosine is derived from ATP release originating from astrocytes, and the effects of diurnal time and SD are expressed in transgenic dnSNARE mice that selectively inhibit exocytosis from astrocytes. The iNOS dependence of this enhanced astrocytic release of ATP following SD has not yet been examined.

Our data therefore suggest that the mechanisms of adenosine release and its diurnal control vary between brain regions. This regional specificity of adenosine release may present a means by which sleep-sensitive areas of the brain can react selectively to wakefulness to control sleep pressure that does not necessarily influence adenosine availability across the whole brain.

### Conclusions

Our study has the following important conclusions. Firstly, the mechanisms of adenosine release (both basal and activity dependent) measured *in vitro* depend on diurnal time and sleep status of the animal prior to sacrifice. Secondly, in the BFB, induction of iNOS is a key step in the elevation of adenosine release from sleep deprivation (activity dependent and basal) and constitutes the biochemical memory of sleep-deprivation in this brain area. Thirdly, adenosine release varies with brain region and different mechanisms of regulation according to diurnal time and sleep status appear to pertain to different regions. Finally, adenosine release in this model is evoked by only some specific excitatory stimuli and is not dependent on calcium.
